# Comparison of Land, Water, and Energy Requirements of Lettuce Grown Using Hydroponic *vs.* Conventional Agricultural Methods

**DOI:** 10.3390/ijerph120606879

**Published:** 2015-06-16

**Authors:** Guilherme Lages Barbosa, Francisca Daiane Almeida Gadelha, Natalya Kublik, Alan Proctor, Lucas Reichelm, Emily Weissinger, Gregory M. Wohlleb, Rolf U. Halden

**Affiliations:** 1 School of Sustainable Engineering and the Built Environment, Arizona State University, Tempe, AZ 85287-5904, USA; E-Mails: glagesba@asu.edu (G.L.B.); falmeida@asu.edu (F.D.A.G.); Natalyaemmanuely.Mohrkublik@asu.edu (N.K.); ajprocto@asu.edu (A.P.); lreichel@asu.edu (L.R.); eweissin@asu.edu (E.W.); gwohlleb@asu.edu (G.W.); 2 Center for Environmental Security, The Biodesign Institute, Global Security Initiative, Arizona State University, 781 E. Terrace Mall, Tempe, AZ 85287-5904, USA

**Keywords:** agriculture, hydroponics, sustainability, water, energy, land use, lettuce, Arizona

## Abstract

The land, water, and energy requirements of hydroponics were compared to those of conventional agriculture by example of lettuce production in Yuma, Arizona, USA. Data were obtained from crop budgets and governmental agricultural statistics, and contrasted with theoretical data for hydroponic lettuce production derived by using engineering equations populated with literature values. Yields of lettuce per greenhouse unit (815 m^2^) of 41 ± 6.1 kg/m^2^/y had water and energy demands of 20 ± 3.8 L/kg/y and 90,000 ± 11,000 kJ/kg/y (±standard deviation), respectively. In comparison, conventional production yielded 3.9 ± 0.21 kg/m^2^/y of produce, with water and energy demands of 250 ± 25 L/kg/y and 1100 ± 75 kJ/kg/y, respectively. Hydroponics offered 11 ± 1.7 times higher yields but required 82 ± 11 times more energy compared to conventionally produced lettuce. To the authors’ knowledge, this is the first quantitative comparison of conventional and hydroponic produce production by example of lettuce grown in the southwestern United States. It identified energy availability as a major factor in assessing the sustainability of hydroponics, and it points to water-scarce settings offering an abundance of renewable energy (e.g., from solar, geothermal, or wind power) as particularly attractive regions for hydroponic agriculture.

## 1. Introduction

Conventional agricultural practices can cause a wide range of negative impacts on the environment. “Conventional” or “modern industrial agriculture” has been historically defined as the practice of growing crops in soil, in the open air, with irrigation, and the active application of nutrients, pesticides, and herbicides. Some of the negative impacts of conventional agriculture include the high and inefficient use of water, large land requirements, high concentrations of nutrients and pesticides in runoff, and soil degradation accompanied by erosion [1,2]. As the world population continues to grow at a rapid rate, so too must the food production. However, approximately 38.6% of the ice-free land and 70% of withdrawn freshwater is already devoted to agriculture [3,4]. To sustainably feed the world’s growing population, methods for growing food have to evolve.

The benefits of hydroponic agriculture are numerous. In addition to higher yields and water efficiency, when practiced in a controlled environment, hydroponic systems can be designed to support continuous production throughout the year [5]. Hydroponic systems are very versatile and can range from rudimentary backyard setups to highly sophisticated commercial enterprises. Various commercial and specialty crops can be grown using hydroponics including tomatoes, cucumbers, peppers, eggplants, strawberries, and many more. Leafy vegetables, such as lettuce can also be grown hydroponically and perform best using the nutrient film technique (NFT) [6]. Hydroponic NFT production involves the circulation of a nutrient solution through shallow channels in a closed-loop system [7].

In 2012, in terms of production by weight, head lettuce was the second largest vegetable crop in the Unites States, second only to onions [8]. A substantial portion of that production (approximately 29% in 2012) occurs in Arizona, primarily in Yuma [8,9]. Since Arizona devotes approximately 69% of its current freshwater withdrawals to agriculture [10], investigations into hydroponic alternatives could be beneficial in reducing the strain on water resources in such regions. There is considerable research available regarding conventional lettuce production and hydroponic lettuce production separately, but few studies that have compared the resource inputs of the two at a commercial level.

Regarding conventional lettuce production, in 2001, the University of Arizona Cooperative Extension developed county-specific crop budgets estimating the operating and ownership costs of producing vegetables in Arizona using representative cropping operations and such resource inputs as water, fuel, and fertilizer [11]. Realizing that the water and energy use for agriculture is substantial, Ackers *et al.* (2008) [12] performed an “order of magnitude” study and determined a reasonable range of estimates for resources used in the production of Arizona agriculture.

Regarding hydroponics, the Ohio State University developed an enterprise model designed to estimate the revenue, expenses, and profitability associated with a typical hydroponic greenhouse lettuce production system in Ohio [13]. Various other authors have investigated components of hydroponic lettuce production as it relates to water and energy inputs [14,15].

The objective of this study is to determine whether hydroponic lettuce production is a suitable and more sustainable alternative to conventional lettuce production in Arizona. For this study, “a suitable and more sustainable alternative” is one that outperforms (*i.e.*, is more efficient than) conventional agriculture in the metrics of land use, water use, and energy use, normalized by yield.

## 2. Experimental Section

Metrics for land, water, and energy use were developed for conventional lettuce production and hydroponic lettuce production using a southwestern Arizona location as a case study scenario. Data related to conventional lettuce production was obtained from crop budgets and governmental agricultural statistics, while data regarding the hydroponic production of lettuce was developed using engineering equations and values reported in literature which were then applied to a hypothetical 815 square meter enclosed hydroponic greenhouse located in Yuma, Arizona. This greenhouse was assumed to be using the nutrient film technique of growing hydroponic lettuce, have temperature controls, supplemental artificial lighting, and water circulation pumps.

### 2.1. Lettuce Grown Using Conventional Agricultural Methods

#### 2.1.1. Conventional Yield

The average yield of head lettuce per acre in Arizona was quantified by taking the average of the past ten years of agricultural census data (2003–2012) that was available from the National Agricultural Statistics Service (NASS) for the United States Department of Agriculture (USDA) [8,16,17,18,19]. The resulting value was then converted into units of kilograms per square meter per year (kg/m^2^/y).

#### 2.1.2. Conventional Water Usage

The water usage related to the conventional production of head lettuce in Arizona was estimated using county-specific crop budgets developed by the University of Arizona Cooperative Extension. Specifically, the two Yuma County crop budgets for head lettuce were used to produce an average estimate of irrigation use of 965 L per square meter per crop [11]. The average value was then converted to liters per kilogram per year (L/kg/y) using the estimated average yield value for conventional lettuce and assuming one crop of lettuce per year.

#### 2.1.3. Conventional Energy Usage

Energy use related to the conventional production of head lettuce in Arizona was limited to the direct use of fossils fuels by farms during operations and the use of electricity in the pumping of irrigation water. An average estimate of fuel use was derived from the Yuma County crop budgets for head lettuce production developed by the University of Arizona Cooperative Extension [11]. The average value was then converted to kilojoules per kilogram per year (kJ/kg/y) using the estimated average yield value for conventional lettuce and assuming one crop of lettuce per year. It was conservatively assumed that all farms require energy to pump irrigation water and that all of the pumps use electricity.

Using an estimate of 1037 kilowatt-hours (kWh) per acre foot pumped [12], a conversion factor of 3600 kilojoules per kWh, the average estimate of water use calculated previously, and the estimated average yield value for conventional lettuce, an average estimate of energy use for the pumping of irrigation water was computed in units of kJ/kg/y.

Together, the average estimates of energy use related to fossil fuels and the pumping of irrigation water were combined to produce an overall average estimate of energy use for the conventional production of head lettuce in Arizona in units of kJ/kg/y.

### 2.2. Lettuce Grown Using Hydroponic Methods

#### 2.2.1. Hydroponic Yield

The average yield of head lettuce grown using hydroponic methods was estimated by evaluating values reported in available literature. Databases searched included; AGRICOLA, Web of Science, Google Scholar, and Science Direct, using keywords “hydroponics” and “head lettuce.” Only publications that contained the necessary information regarding yield, type of hydroponic system (e.g., NFT), configuration, production area, and greenhouse area were used. Once the information was gathered, the data was standardized.

Configurations from three studies were averaged to get an approximate plant density of 24 plants per square meter of greenhouse [7,13,20]. With supplemental artificial lighting and temperature controls lettuce production can be continuous year round, with a full harvest cycle completed every 30 days (or approximately 12 harvests per year) [13]. The mass of edible lettuce per plant was averaged from two sources to get an average mass of 144.6 grams per plant [13,21]. Using the estimates for plant density, number of harvests, and plant mass, the average yield of hydroponic lettuce per year was calculated in units of kg/m^2^/y.

#### 2.2.2. Hydroponic Water Usage

The water usage related to the hydroponic production of head lettuce in Arizona was estimated by considering the average evapotranspiration of each individual plant, which takes into account the water loss due to evaporation and transpiration. Both (2014) [22] reported that lettuce plants grown hydroponically must transpire at least 400 mL per gram of dry mass accumulated to avoid tipburn damage. Using a ratio of dry mass to fresh mass of 0.045 [22] and the average mass per head of lettuce calculated previously, the average evapotranspiration per plant was calculated. This value was then conservatively increased by an additional 10% to account for periodic flushing of the nutrient solution. This estimate was used in combination with the estimated yield values for hydroponic lettuce to create an average estimate of water usage in units of L/kg/y.

#### 2.2.3. Hydroponic Energy Usage

Energy use related to the hydroponic production of head lettuce in Arizona focused on the energy demands associated with supplemental artificial lighting, water pumps, and heating and cooling loads.

The energy use related to supplemental artificial lighting was determined by assuming a 24-h photoperiod and a recommended daily Photosynthetically Active Radiation (PAR) of 17 mol/m^2^/day of both natural and supplemental light [5], for optimal lettuce production. It was also assumed that half of the required radiation could be obtained from natural lighting. Assuming a wavelength of 600 nm (the approximate average wavelength of the high pressure sodium bulbs used in supplemental light), the following equation was used to calculate the energy of a mole of photons:

(1) $$E=\;\frac{h\times c}{\text{λ}}\;\times \frac{6.022\;\times \;10^{23}}{mol}$$

where

E: Energy per mole of photons (J/mol)
h: Planck’s constant (6.626 × 10^−34^ J·s)
c: Speed of light (2.998 × 10^8^ m/s)
λ: Wavelength of light (m)

The resulting value was then used in conjunction with the estimated yield values for hydroponic lettuce to calculate the energy demand from supplemental lighting in units of kJ/kg/y.

The energy use related to the pumping of water associated with the hydroponic production of lettuce was estimated using an average pumping time and a representative pump power rating. Lopes da Luz *et al.* (2008) [15] performed a study indicating that the optimal irrigation regimen for hydroponic lettuce resulted in four hours thirty minutes of pumping per day. Regarding the pump power rating, a study [23] suggested that a 0.5 horsepower (HP) pump is sufficient to deliver water to a hydroponic system with an average of 2040 heads of lettuce. Together, the average horsepower per plant along with the average pumping time per day was used in conjunction with the estimated yield values for hydroponic lettuce to estimate the energy use related to pumping in units of kJ/kg/y.

The energy use related to heating and cooling loads associated with the hydroponic production of lettuce was estimated by calculating the design heat load of a hypothetical greenhouse in Arizona. The following equation, based on standard heat transfer equations, was used to determine these values [24].

(2) $$Q=U\times SA\times (T_{in}-T_{out})$$

where,

Q: Heat lost or gained due to outside temperature (kJ·h^−1^)
U: Overall heat transfer coefficient (kJ·h^−1^·m^−2^·°C^−1^)
SA: Surface area of greenhouse (m^2^)
T_in_: Inside air set point temperature (°C)
T_out_: Outside air temperature (°C)

For the purpose of these estimations, 23.9 °C was used as the set point temperature of the greenhouse, as this is the optimal temperature for lettuce cultivation [5]. Polyethylene is one of the most common materials used in greenhouse construction, and the overall heat transfer coefficient for this material (24.5 kJ/h/m^2^/°C) was used in calculations [24]. The heating and air conditioning efficiency was conservatively estimated to be 75% and the ceilings were estimated to be 3.0 m high. The dimensions of the hypothetical greenhouse were estimated to be 63.7 m by 12.8 m. For comparison purposes, it was assumed that the hydroponic greenhouse would be located in Yuma, Arizona, the same location where the majority of non-hydroponic head lettuce in Arizona is produced [9]; therefore, the average monthly temperatures for this region were used to estimate the temperatures external to the greenhouse [25]. The final energy estimates were used in conjunction with the estimated yield values for hydroponic lettuce to create a metric in units of kJ/kg/y.

Together, the estimates of energy use related to supplemental artificial lighting, water pumps, and heating and cooling loads were combined to produce an overall estimate of energy use for the hydroponic production of head lettuce in Arizona in units of kJ/kg/y.

## 3. Results and Discussion

**Figure 1:**
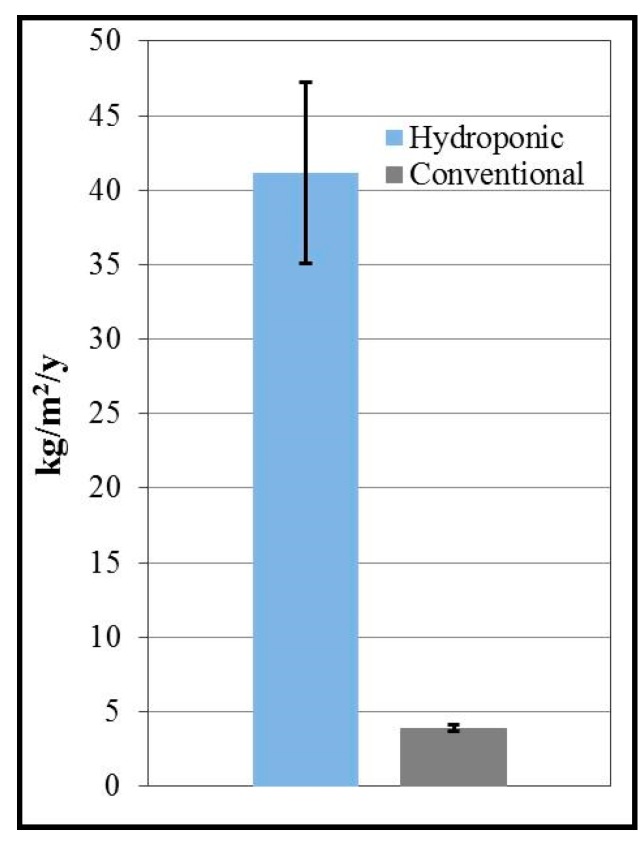
Modeled annual yield in kilograms per square meter of lettuce grown in southwestern Arizona using hydroponic *vs.* conventional methods (Error bars indicate one standard deviation).

In terms of yield per area, the hydroponic production of lettuce in Arizona was found to be 11 ± 1.7 times greater than that of its conventional equivalent. Specifically, hydroponic lettuce production was calculated to result in a yield of 41 ± 6.1 kg/m^2^/y (±standard deviation, SD, here and in the following), while conventional lettuce production was projected to yield 3.9 ± 0.21 kg/m^2^/y (Figure 1).

**Figure 2:**
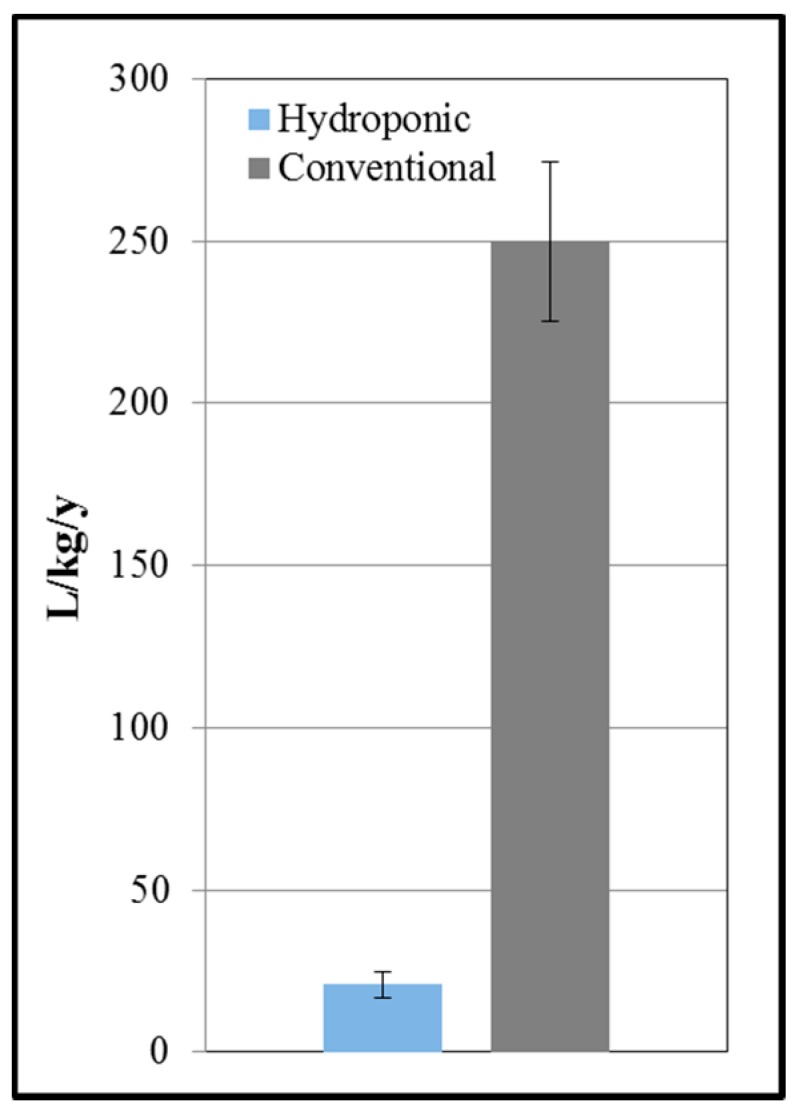
Modeled annual water use in liters per kilogram of lettuce grown in southwestern Arizona using hydroponic *vs.* conventional methods (Error bars indicate one standard deviation).

Water consumption between the hydroponic and conventional production of lettuce in Arizona was comparable on an area basis, but when normalized by yield the average was 13 ± 2.7 times less water demand in hydroponic production compared to conventional production. Specifically, hydroponic lettuce production had an estimated water demand of 20 ± 3.8 L/kg/y, while conventional lettuce production had an estimated water demand of 250 ± 25 L/kg/y (Figure 2).

**Figure 3:**
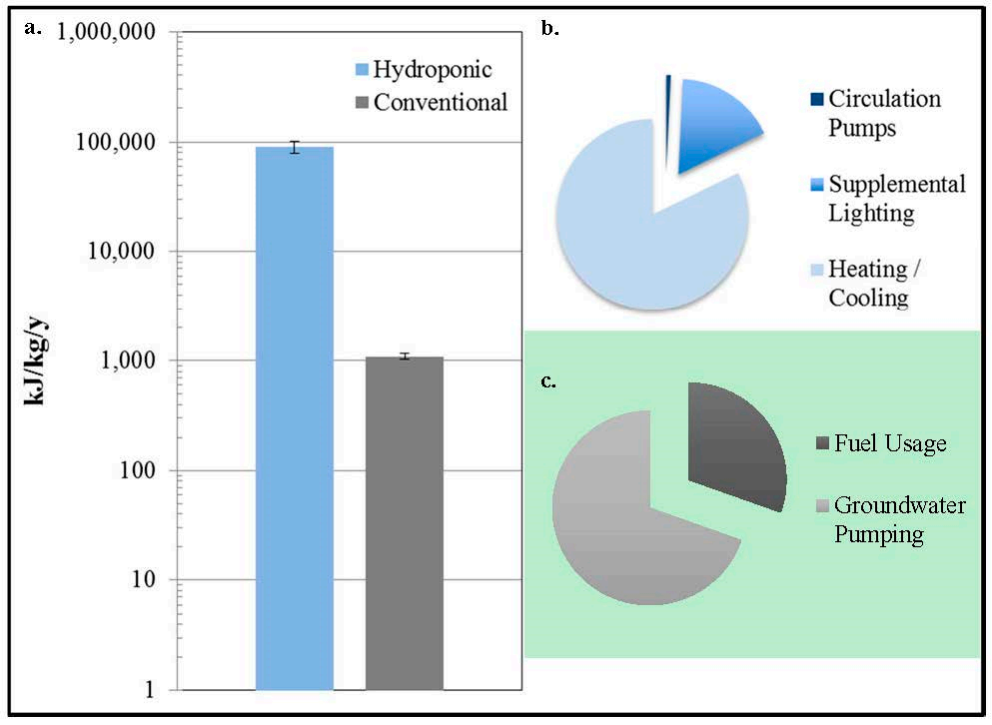
(**a**) Modeled annual energy use in kilojoules per kilogram of lettuce grown in southwestern Arizona using hydroponic *vs.* conventional methods; (**b**) The energy use breakdown related to the hydroponic production of lettuce; (**c**) The energy use breakdown related to the conventional production of lettuce (Error bars indicate one standard deviation).

Results for energy consumption found that the hydroponic production of lettuce in Arizona requires 82 ± 11 more energy per kilogram produced than the conventional production of lettuce in Arizona. Dominating the hydroponic energy use are the heating and cooling loads at 74,000 ± 10,000 kJ/kg/y, followed by the energy used for the supplemental artificial lighting at 15,000 ± 2100 kJ/kg/y. The circulating pumps contributed the least to the total energy use at 640 ± 120 kJ/kg/y. In total, the hydroponic energy use was calculated to equal 90,000 ± 11,000 kJ/kg/y (Figure 3).

The total energy use for the conventional production of lettuce in Arizona was calculated to be 1100 ± 75 kJ/kg/y (Figure 3). This total was split between the energy use related to fuel usage at 330 ± 20 kJ/kg/y and groundwater pumping at 760 ± 74 kJ/kg/y.

The objective of this study was to determine whether hydroponic lettuce production is a suitable and more sustainable alternative to conventional lettuce production in Arizona. According to the results presented, summarized in Table 1, while the hydroponic production of lettuce results in higher yields and more efficient water use, the controlled environment from which the hydroponic system produces its higher yields exhibits a higher energy demand.

**Table 1:** Summary of modeled annual data with standard deviations (S.D.).

Production Method	Yield (kg/m^2^/y)		Water Use (L/kg/y)		Energy Use (kJ/kg/y)	
	Value	S.D.	Value	S.D.	Value	S.D.
Conventional	3.9	0.21	250	25	1100	75
Hydroponics	41	6.1	20	3.8	90,000	11,000

Higher yields of hydroponics result from the controlled environmental conditions maintained within the hydroponic greenhouse, which allow for continuous production year round. These conditions also promote a reduction in the number of days required for each harvest cycle, allowing for multiple crops per year. This benefit of hydroponic production is not unique to lettuce alone, but will vary depending on the operational parameters under which the crop is grown.

Similarly, most hydroponic systems will utilize water more efficiently than conventional farming. The volume of water consumed per plant in a hydroponic system is not different from that grown using conventional methods; however, the hydroponic system delivers the water more efficiently, with a larger percentage of the water going to plant evapotranspiration [26]. For example, lettuce has shallow roots, but is primarily irrigated through flood furrow irrigation in southwestern Arizona. Water not quickly absorbed by the roots is lost to percolation. Increases in the use of low-flow and more-targeted irrigation techniques could lower the overall water use of conventional farming.

As mentioned previously, most of the energy use for the hypothetical hydroponic greenhouse can be attributed to the heating and cooling loads. This is primarily due to the fact that the greenhouse was sited in Yuma, Arizona, an area which can have average temperatures of 34.7 °C in the summer and 14.1 °C in the winter [25]. For illustrative purposes, the percent heating and cooling energy demand by month is presented in Table 2. Greenhouses located in more moderate climates (*i.e.*, climates closer to the greenhouse set point temperature) will experience a lower energy demand. In fact, in certain climates heating and cooling systems may not be required, but instead replaced by a passive ventilation system, thus reducing the overall energy demand considerably. The feasibility of hydroponic systems is heavily reliant on the climate of farming locations.

**Table 2:** Percent heating and cooling energy demand by month.

Month	January	February	March	April	May	June	July	August	September	October	November	December
Average Temperature (°C)	14.8	16.6	19.6	22.8	27.4	31.7	34.7	34.6	31.7	25.3	18.7	14.1
Percent Energy Demand	12%	8%	6%	1%	5%	10%	14%	14%	10%	2%	6%	13%

The next highest use of energy for the hypothetical hydroponic greenhouse is for the supplemental artificial lighting, which is used to maximize crop yield and maintain consistent production year round [7]. Some systems use supplemental lighting to create a 24-h photoperiod, especially during the first few days of plant growth, whereas others may use supplemental lighting for only a few hours a day [5,13]. In addition, small and low-output systems may not use artificial lighting at all. This study assumed that maximum yield was desired and did not perform a cost-benefit analysis of reducing or eliminating supplemental lighting. There are various studies that have tried to optimize supplemental lighting systems [14,27,28]. Progress in this area of research could lead to improved energy efficiency for hydroponic lettuce.

Due to the high energy demands, at this time, commercial hydroponics is not a suitable alternative to conventional lettuce production in Yuma, Arizona. One possible way to make commercial hydroponics a more sustainable and suitable alternative would be to relocate the greenhouse to an area where there are cheap and renewable sources of energy, such as solar, geothermal or wind power; though keeping in mind that the initial investment required for such technologies may be cost-prohibitive. For instance, a hydroponic greenhouse could be sited in an area without arable soil, or on previously developed areas with impervious pavement, where land is cheap and could be used for photovoltaic solar panels. Assuming a conversion efficiency of 14% and an average daily solar radiation value of 6.5 kWh per square meter per year [29] the ratio of greenhouse to solar panel area that would be required to offset the full energy demand of the hydroponic system is approximately 1:3.0, with an understanding that the 24-h production cycle along with seasonal and day-to-day changes in solar radiation would require the system to be connected to the electrical grid. This is still an improvement over conventional production, which uses ten times more land and water on a yield basis and requires arable land. Eliminating the need for arable land has other benefits including versatility in system siting and a potential reduction in the distance in which food must travel. Performing a life cycle assessment that considers the environmental impact of food transportation could show whether this benefit of hydroponics is significant.

An alternative scenario to consider is one in which much of the energy-intensive characteristics of advanced and commercial hydroponic operations are abandoned in favor of simpler systems, a practice known as “simplified hydroponics” or “popular hydroponics.” These operational changes come at the cost of total yield; however, such systems can still out-perform conventional systems by a factor of three to four on an area basis [30]. The Food and Agriculture Organization of the United Nations maintains that such systems can be as small as one square meter in size, but that most household simplified hydroponic gardens range between 10 to 20 square meters in size. Communities have also adopted simplified hydroponic gardens at sizes greater than 200 square meters—a scale in which it becomes viable to sell excess produce for income [30].

There are several limitations to this study that need to be considered. To begin, the calculations for the conventional production of lettuce assumed that only one crop of lettuce can be grown in a given year. While this might be true, much of the land used to produce lettuce is dedicated to warm season crops when lettuce is not in production. It’s possible that these crops have water and energy requirements that differ from lettuce. Additionally, the hydroponic greenhouse was assumed to be of commercial scale and was optimized for maximum production. Furthermore, as seen in Equation (2), the greenhouse was assumed to be of a specific size and located in Yuma, Arizona (one of the most productive areas for lettuce grown conventionally in the United States). In reality, hydroponic greenhouses come in a variety of configurations and can be located almost anywhere. As such, it would be reasonable to model multiple hypothetical greenhouses. Changing the assumptions surrounding the hypothetical greenhouses could produce alternative results. In addition, this study solely focused on direct energy inputs to hydroponic and conventional lettuce production and did not consider energy embodied in chemical or material inputs. Performing a full life cycle assessment of hydroponics *vs.* conventional lettuce production could also produce alternative results, with labor hours potentially figuring prominently into the economic equation. Lastly, this study did not discuss additional factors that might inhibit the successful implementation of hydroponics, such as energy scarcity and higher upfront capital costs.

## 4. Conclusions

Despite its high demand for energy, hydroponics remains a promising technology. Several factors could influence the feasibility of hydroponic production of crops, specifically lettuce, in the future. As more sophisticated control devices become available, the cost of maintaining the controlled environment of hydroponic greenhouses could decrease. The future availability of water, land, and food will also influence feasibility through increased demand. Increasing land and water scarcity will make the more land- and water-efficient hydroponic systems more appealing to city planners. Government and local grass-roots support could also influence the future of hydroponic farming, as subsidies could be used to offset the high initial cost of hydroponic infrastructure or more simplified hydroponic systems take hold.

At this point in time, hydroponic farming of lettuce cannot be deemed a more sustainable alternative to conventional lettuce farming techniques, but it provides promising concepts that could lead to more sustainable food production. In summary, hydroponic gardening of lettuce uses land and water more efficiently than conventional farming and could become a strategy for sustainably feeding the world’s growing population, if the high energy consumption can be overcome through improved efficiency and/or cost-effective renewables.
